# Effects of exercise combined with different dietary interventions on cardiovascular health a systematic review and network meta-analysis

**DOI:** 10.1186/s12872-025-04666-z

**Published:** 2025-03-26

**Authors:** Yang Hei, Yongchao Xie

**Affiliations:** 1 Centre for Sport Nutrition and Health, Centre for Nutritional Ecology, School of Physical Education (Main Campus), Zhengzhou University, Zhengzhou, 450001 China; 2 Department of Physical Education, College of Education, Seoul National University, Seoul, 08826 South Korea

**Keywords:** Exercise, Caloric restriction, Intermittent fasting, Ketogenic diet, Cardiovascular Health

## Abstract

**Background:**

Numerous studies have shown that exercise and dietary interventions positively impact CVD outcomes; however, there is substantial variability in the efficacy of different interventions. The absence of direct comparisons between multiple interventions complicates the determination of their relative effects. This study aims to synthesize the literature on the impacts of exercise, dietary, and combined interventions on cardiovascular health indicators, and to perform a network meta-analysis to rank the efficacy of these approaches, providing a theoretical foundation for selecting optimal intervention strategies.

**Methods:**

We systematically reviewed the literature from database inception through September 2024, searching PubMed, Web of Science, Embase, and the Cochrane Library. Data were aggregated and analyzed using network meta-analysis, with intervention efficacy ranked according to Surface Under the Cumulative Ranking (SUCRA) curves.

**Results:**

The efficacy of these interventions was ranked as follows: 1). Triglycerides (TG) Reduction: CR + EX > CR > 5/2F + EX > TRF + EX > KD > 5/2F > KD + EX > EX > CON > TRF. 2). Total Cholesterol (TC) Reduction: CR + EX > CR > 5/2F + EX > 5/2F > TRF + EX > EX > CON > KD > TRF > KD + EX. 3). High-Density Lipoprotein (HDL) Increase: 5/2F > KD > KD + EX > TRF + EX > CON > EX > TRF > 5/2F + EX > CR + EX > CR. 4). Low-Density Lipoprotein (LDL) Reduction: CR + EX > CR > TRF + EX > KD + EX > EX > KD > 5/2F > CON > 5/2F + EX > TRF. 5). Systolic Blood Pressure (SBP) Reduction: 5/2F > CR + EX > CR > EX > TRF > TRF + EX > CON > 5/2F + EX. 6). Diastolic Blood Pressure (DBP) Reduction: CR > CR + EX > TRF > 5/2F > TRF + EX > EX > CON > 5/2F + EX.

**Conclusion:**

CR and CR + EX demonstrated the most positive effects on cardiovascular health indicators. In contrast, 5/2F + EX ranked relatively low in effectiveness, with its impact on several indicators being even lower than that of CON.

**Supplementary Information:**

The online version contains supplementary material available at 10.1186/s12872-025-04666-z.

## Introduction

Data from the World Health Organization (WHO) indicate that cardiovascular disease (CVD) is the leading cause of death and disability worldwide [1]. The number of global CVD cases rose from 271 million in 1990 to 523 million in 2019 [2]. In 2021 alone, ischemic heart disease accounted for 9.44 million deaths, while stroke caused 3.87 million deaths [3]. A 20-year community surveillance study in the United States showed a shift in CVD incidence toward younger populations, corresponding with an increase in cardiovascular risk factors among younger patients [4]. The growing number of CVD cases places a significant economic burden on healthcare systems worldwide, making the mitigation of this trend an urgent priority.

CVD encompasses a range of conditions, including coronary artery disease, heart failure, stroke, and hypertension [5], all of which share a common underlying mechanism: atherosclerosis (AS) [6]. AS is a critical predictor of CVD and is considered a major precursor to its development [7]. According to the American Heart Association's Life's Essential 8 (LE8) score, blood pressure and blood lipids are key indicators of cardiovascular health [8]. Hypertension and dyslipidemia damage the vascular endothelium, promoting the development of AS, which ultimately leads to CVD [9, 10]. Dyslipidemia is characterized by elevated serum triglycerides (TG), total cholesterol (TC), and low-density lipoprotein (LDL), along with reduced high-density lipoprotein (HDL) [11]. Hypertension exacerbates endothelial damage, and in the presence of dyslipidemia, it increases oxidative stress in the vasculature, leading to inflammation, impaired endothelium-dependent vasodilation, and the promotion of atherosclerotic plaque formation [12]. This can result in the rupture of atherosclerotic plaques or coronary endothelial erosion, ultimately precipitating CVD [13].

Oral antihypertensive medications are the most common treatment for hypertension, primarily working by regulating dysfunction in the renin–angiotensin–aldosterone system [14]. However, long-term use of these medications can place significant strain on the kidneys and liver and may lead to dependency and side effects [15, 16]. Furthermore, hypertension often recurs once medication is discontinued. As a result, the pursuit of non-pharmacological methods for alleviating and treating hypertension has become a key objective. In the early twentieth century, the WHO recommended exercise as an intervention to reduce hypertension [17]. The American College of Sports Medicine has also stated that exercises such as yoga, Pilates, and Tai Chi have been proven to reduce blood pressure [18]. Other studies have shown that both resistance training and aerobic exercise can effectively help manage hypertension [19, 20]. A meta-analysis of 64 randomized controlled trials found that both aerobic and resistance exercises significantly reduced blood pressure [21]. In terms of dietary interventions, much research has focused on the Mediterranean diet and the Dietary Approaches to Stop Hypertension. This network meta-analysis, however, emphasizes the effects of energy metabolism on cardiovascular health, rather than the impact of specific nutrients. Therefore, the dietary interventions included in this study are limited to Calorie Restriction (CR), Time-Restricted Feeding (TRF), 5/2 Intermittent Fasting (5/2F), and Ketogenic Diet (KD). Substantial evidence indicates that CR can help lower blood pressure and improve hypertension, with calorie-restricted diets significantly reducing both systolic blood pressure (SBP) and diastolic blood pressure (DBP) in both animal and human studies [22–27]. This may be due to the fact that CR reduces oxidative stress levels in the heart, thereby decreasing the conversion of nitric oxide (NO) to peroxynitrite in the endothelium, leading to a blood pressure-lowering effect [28, 29]. Other studies have demonstrated that CR provides vascular protection by lowering blood pressure and improving endothelial function. Potential mechanisms include enhanced expression and activity of endothelial nitric oxide synthase (eNOS), as well as increased NO production [30–32]. Additionally, in a diet-induced obesity mouse model, CR has been shown to reverse endothelial dysfunction, reduce vascular oxidative stress in rats, and lower blood pressure [33–35]. Several effects of CR may be mediated through AMP-activated protein kinase (AMPK) activation, such as improving vascular compliance and lowering blood pressure in hypertensive rats, or reducing endothelial lipotoxicity [36–38]. One study found that CR had beneficial effects on reducing blood pressure and regulating pro-inflammatory cytokines in tissues, CR mitigates the decline in anti-inflammatory capacity associated with obesity, promotes the levels of vascular endothelial growth factor, and thereby contributes to the reduction of blood pressure [39]. Another study showed that a very low-calorie ketogenic diet could safely lower blood pressure in obese and hypertensive women [40]. The inflammatory state induced by a ketogenic diet is a major predictor of changes in blood pressure. Chronic inflammation, commonly associated with obesity, increases the production of ROS, leading to oxidative stress and endothelial dysfunction. This results in the loss of normal vascular tone and structure. Furthermore, as inflammation persists, the bioavailability of NO decreases, impairing its primary function as a vasodilator and hindering vascular relaxation and dilation [41]. However, a meta-analysis of 23 studies indicated that, despite evidence suggesting KD may offer some health benefits, it does not appear to be an appropriate dietary intervention for lowering blood pressure [42]. These findings underscore the significant variability in the effects of different exercise and dietary interventions on blood pressure.

Regarding hyperlipidemia, the American College of Cardiology identifies LDL, HDL, TC, and TG as key risk factors influencing the incidence of CVD events. Studies have shown that physical exercise can help prevent and manage hyperlipidemia, obesity, overweight, and hypertension, making it an effective intervention for reducing CVD risk [43, 44]. Additionally, research suggests a positive dose–response relationship between exercise load and intensity with plasma HDL levels, while a negative correlation exists with TG levels [45]. A meta-analysis of 148 randomized controlled trials found that exercise significantly improves TC, TG, HDL, and LDL levels [46]. In terms of dietary interventions, one study showed that CR significantly reduced waist circumference, fat mass, SBP, DBP, and BMI, while also effectively lowering fasting glucose, insulin, insulin resistance, TG, TC, LDL, and HDL in metabolic parameters [47, 48]. Another study on CR found that it could significantly reduce TC, LDL, the TC/HDL ratio, TG, fasting glucose, fasting insulin, high-sensitivity C-reactive protein, and platelet-derived growth factor AB, as well as SBP and DBP, while HDL levels were higher than those in the normal diet group [49]. CR is gaining popularity as a treatment option for various pathological conditions, including dyslipidemia, as reducing calorie intake can decrease endogenous cholesterol production. However, one meta-analysis highlighted ongoing controversy regarding CR’s effectiveness in lowering blood lipids and increasing HDL levels [50]. For intermittent fasting, one study found that short-term fasting (approximately 15–16 h) significantly reduced SBP and DBP, with trends suggesting decreases in TC, LDL, and HDL, though TG levels showed an upward trend [51]. Another study on TRF suggested that fasting overnight after morning feeding could increase HDL concentrations and decrease TG levels [52]. However, a meta-analysis found no statistically significant differences in SBP, DBP, TC, TG, LDL, HDL, fasting glucose, or fasting insulin between the intermittent fasting group and the control group [53]. Regarding the KD, one study involving 20 normolipidemic, normal-weight men showed that a 6-week ketogenic diet significantly reduced fasting serum triglycerides and postprandial lipid levels, while increasing HDL [54]. However, other reports indicated that a 12-month ketogenic diet significantly increased serum TC, LD, and TG levels [55–57], making KD’s impact on lipid levels uncertain. One study may help explain this uncertainty, showing that a notable side effect of KD is a sharp rise in TG and LDL levels during the initial months. However, the increase in LDL was accompanied by an increase in the size and volume of LDL particles, which may reduce the likelihood of LDL-induced atherosclerosis [58]. However, dietary intervention also has certain side effects. The CR group may experience a reduction in lean body mass, while the KD group may enter ketosis within 30–45 days. All dietary intervention groups may encounter side effects during the initial phase, including irritability, difficulty concentrating, and reduced energy levels. However, these symptoms typically improve as the body gradually adapts to the intervention. Regarding combined interventions of exercise and diet, one study indicated that dietary interventions combined with regular physical activity improved various body composition and metabolic indicators, except for HDL and glucose levels [59]. A study on the 5/2F found that 3 months of 5/2F + EX significantly reduced plasma TC and TG levels [60].

Regarding the combined intervention of diet and exercise, previous meta-analyses have demonstrated that both exercise and dietary interventions positively influence lipid profiles, glycemic markers, and blood pressure [61–66]. More recent studies have shown that the combination of exercise and diet can more effectively improve cardiovascular health indicators than either intervention alone [67, 68]. However, a meta-analysis examining the effects of EX and intermittent fasting (IF) on body composition and cardiometabolic health, which included 11 studies, suggested that while the combination of EX and IF resulted in greater improvements in body composition compared to EX or IF alone, it did not lead to additional benefits in cardiometabolic health markers. Thus, while the combined intervention may be effective for weight and fat reduction, it does not appear to offer synergistic effects on other cardiometabolic health markers [69]. This highlights the variability in the outcomes of different dietary interventions and suggests that combining dietary interventions with exercise does not necessarily result in cumulative benefits.

Most previous research has focused on comparing two or three interventions, without conducting horizontal comparisons across multiple approaches. This has made it difficult to determine differences in their effectiveness. Therefore, this study synthesizes the existing literature on exercise, dietary, and combined interventions and employs a network meta-analysis to rank the effectiveness of different approaches on cardiovascular health indicators. The goal is to provide a theoretical basis to help individuals make informed decisions when choosing intervention strategies.

## Methods

### Protocol and registration

This systematic review was conducted in accordance with the Preferred Reporting Items for Systematic Reviews and Meta-Analyses (PRISMA) guidelines [70]. The review protocol has been registered in PROSPERO (identifier: CRD42024586866).

### Search strategy

A thorough search was performed across PubMed, Web of Science, Embase, and the Cochrane Library from their inception to September 2024. Only English-language publications were considered. Two authors (Y.X. and Y.H.) independently screened and assessed the articles according to pre-established inclusion criteria. Discrepancies in selection decisions were resolved through discussion. The specific search terms can be found in Supplementary File S1.

### Inclusion criteria and exclusion criteria

#### Inclusion criteria

1. Randomized controlled trials (RCTs) that include at least two groups receiving exercise, dietary intervention, or a combination of these interventions.
2. Participants must be adults aged 18 years or older.
3. Participants must be healthy individuals.
4. Outcome measures must include at least one of the following: TG, TC, HDL, LDL, SBP, or DBP.

#### Exclusion criteria

1. Studies that are not randomized controlled trials.
2. Studies involving non-human subjects.
3. Non-original research, including reviews, letters, case reports, or studies with incomplete or unclear data.
4. Studies involving children or individuals with pre-existing conditions

### Data extraction

Before data extraction, a table was designed to record the relevant information to be collected. The data extracted from the included studies consists the study title, participants' age, health status, type of exercise, exercise frequency, intensity, duration, and quality assessment. Two authors (Y.H. and Y.X.) independently performed the data extraction, with any discrepancies resolved through discussion and mutual consultation. For studies presenting data in graphical form rather than numerical format, data were extracted using WebPlotDigitizer (https://automeris.io/; accessed September 2024). Following the methodology of previous studies [71], all data formats were converted to "mean ± SD".

### Risk of bias assessment

Two authors (Y.X. and Y.H.) independently assessed the quality of the included RCTs using the Cochrane risk of bias tool, resolving any disagreements through discussion. The assessment methods followed those used in previous studies [71].

### Statistical analysis

The extracted data were analyzed using Review Manager 5.3 and Stata MP 17 software. The analytical steps followed the methodology of previous studies [71], with the addition of cluster ranking diagrams to illustrate the effects of interventions on two factors. The results were summarized using standardized mean differences (SMDs) and 95% confidence intervals (95% CIs).

## Results

### Search results and study selection

**Fig. 1:**
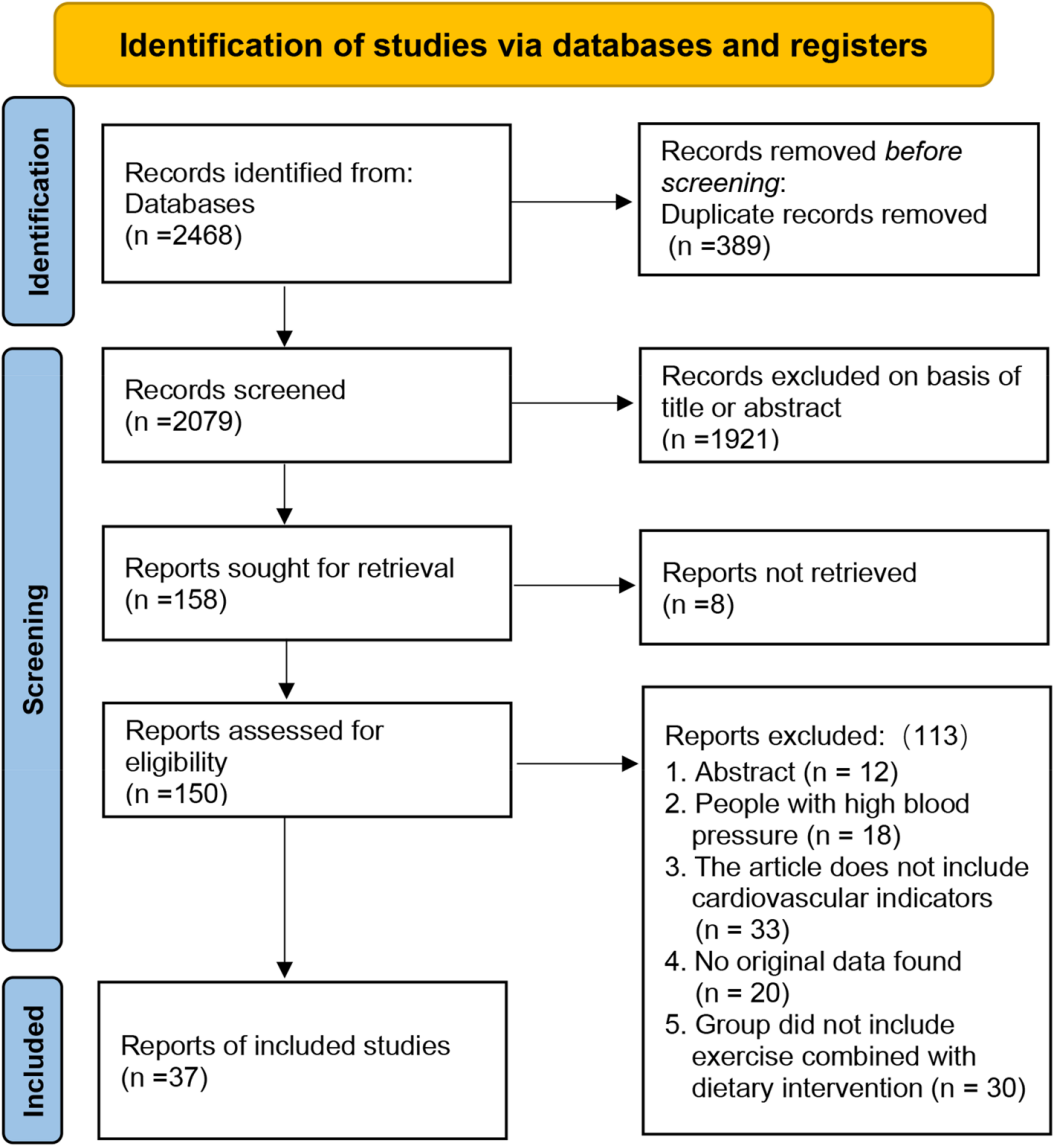
Flow diagram of study selection

As of September 2024, a total of 1,752 articles were retrieved from four databases: PubMed (248), Web of Science (1,644), Embase (322), and the Cochrane Library (254). After removing 389 duplicate articles, 1,921 articles were excluded based on titles or abstracts, leaving 158 articles for full-text review. Before the full-text review, 8 articles could not be located. Following the review, 113 articles were excluded for the following reasons: Abstract only (*n* = 12), Participants had high blood pressure (*n* = 18), Cardiovascular indicators were not included (*n* = 33), No original data provided (*n* = 20), and the study did not include exercise combined with dietary intervention (*n* = 30). Ultimately, 37 articles were included in the final analysis (Fig. 1). During the screening process, the two authors independently summarized the results, yielding a kappa value of 0.7335, which indicates a high level of agreement between them.

### Study characteristics

A total of 37 articles were included in the meta-analysis (Supplementary Table S1). These studies, conducted between 1995 and 2024, involved 2,023 participants aged 18 years and older. The duration of exercise interventions ranged from 3 weeks to 1 year and included both aerobic and resistance training exercises. Dietary interventions included calorie restriction, 5:2 intermittent fasting, time-restricted fasting, and the ketogenic diet. The specific characteristics of the studies are detailed in Supplementary Table S1. Of the included studies, 21 reported on exercise interventions, 17 on calorie restriction, 2 on 5:2 intermittent fasting, 4 on time-restricted fasting, and 2 on the ketogenic diet. Additionally, 19 studies examined the combination of exercise and calorie restriction, 3 studies reported on exercise combined with 5:2 intermittent fasting, 8 studies examined exercise combined with time-restricted fasting, and 5 studies focused on exercise combined with the ketogenic diet. The basal metabolic rate (BMR) of the CR group was measured one week prior to the start of the intervention. During the intervention, the caloric intake of the CR group was reduced by 10%–25% of their BMR daily. The 5/2F and TRF groups were allowed to follow an ad libitum diet during their feeding periods. For the KD group, carbohydrate intake was restricted to less than 5% of their total energy intake. All dietary intervention strategies were developed by professional nutritionists, and in most studies, participants were provided with 1–2 main meals per day or were required to document the types and quantities of food they consumed each day. Among the 37 studies included, the majority focused on common moderate-to-low intensity exercises, such as running, cycling, jumping rope, walking, and gym workouts. The intensity criteria were set to no more than 69% of maximal oxygen uptake, no more than 74% of maximal heart rate, and no more than 69% of maximal heart rate reserve [72]. Exercise duration ranged from 30 to 60 min per session, with a frequency of 2 to 5 sessions per week.

### Risk of bias in included studies

Of the 37 included studies, 3 could not be assessed for risk in random sequence generation, 22 could not be assessed for risk in allocation concealment, and 5 could not be assessed for risk in incomplete outcome data. All evaluated studies were assigned an unclear risk level for the blinding of participants and personnel, as it is unlikely that the dietary and exercise interventions in the experimental groups could be blinded effectively (Supplementary Figures S1 and S2).

### Effects of the interventions

We first conducted an inconsistency analysis. If significant inconsistencies were identified, subgroup analyses were performed based on age, sex, and type of exercise. When agreement was satisfactory, a network diagram was created to explore the distribution of key characteristics across the studies. Subsequently, a ranking table was generated to obtain pairwise comparison data. Finally, a SUCRA plot was produced to determine the ranking of different intervention methods.

#### The effect of different interventions on TG

**Fig. 2:**
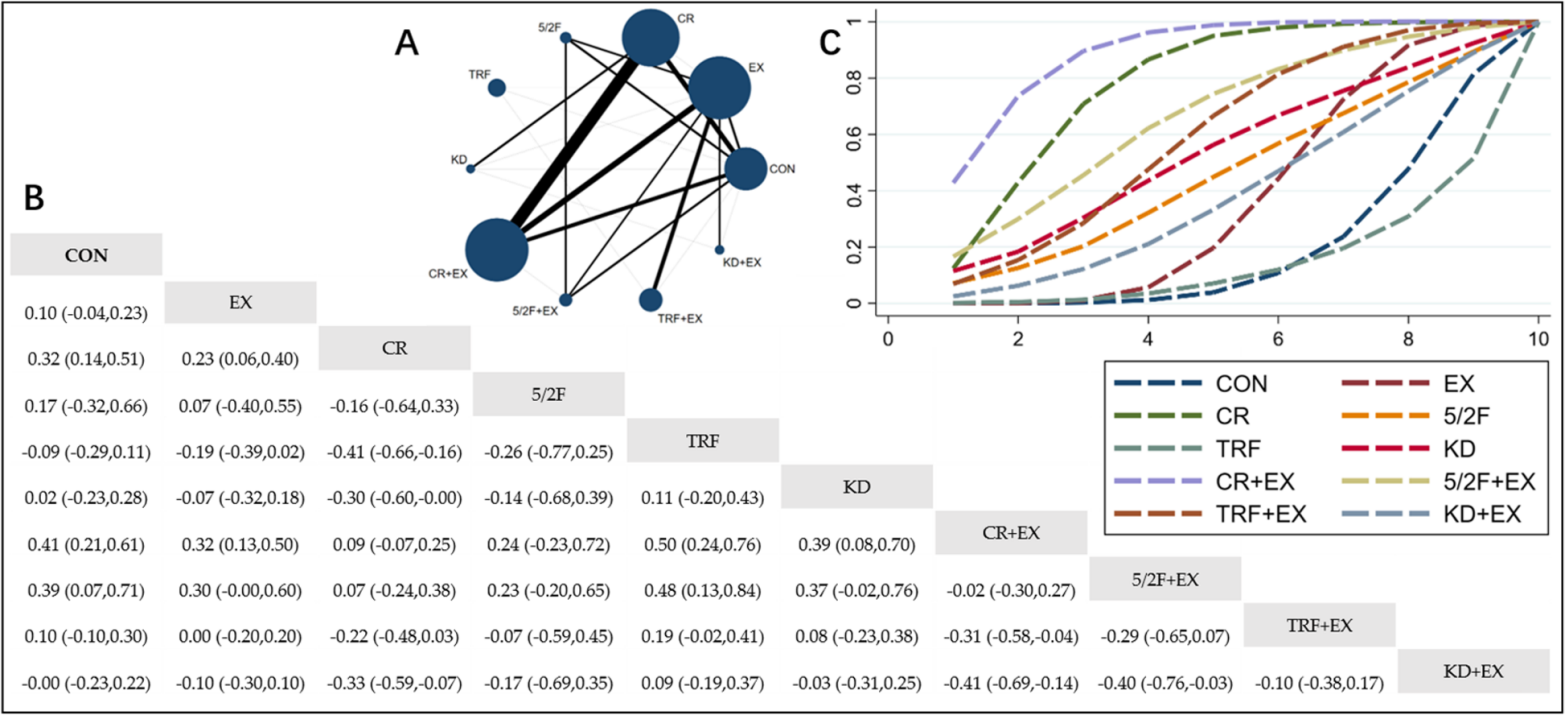
Network Meta-Analysis of TG: Network Plot, League Table, and SUCRA Plot. **A** presents the Network Plot. The size of the nodes is proportional to the sample size of each intervention, and the thickness of the lines corresponds to the number of available studies. **B** displays the pairwise comparison League Table, where the estimated effect size differences (SMD with 95% CI) represent the difference between the intervention on the top and the intervention on the right. **C** illustrates the SUCRA Plot, where the size of the area under the curve indicates the effectiveness of each intervention

The Network Plot, League Table, and SUCRA Plot illustrating the effects of different interventions on TG are presented in Fig. 2.

As shown in Fig. 2A, most of the included studies mainly compared the effects of CR + EX versus CR or EX alone. The groups involving CR + EX, CR, EX, and CON had the largest number of participants. Figure 2B summarizes the estimated effect size differences (SMD with 95% CI) for pairwise comparisons of the 10 intervention methods. Compared to the CON group, the CR group, CR + EX group, and 5/2F + EX group demonstrated a significant reduction in TG levels, while TG reductions in other groups were not significant. Figure 2C presents the SUCRA ranking of the 10 intervention methods, with the area under the curve representing the effectiveness of each intervention. A larger area under the curve indicates a higher ranking of effectiveness. For TG reduction, the ranking of the 10 interventions is as follows: CR + EX > CR > 5/2F + EX > TRF + EX > KD > 5/2F > KD + EX > EX > CON > TRF.

#### The Effect of Different Interventions on TC

**Fig. 3:**
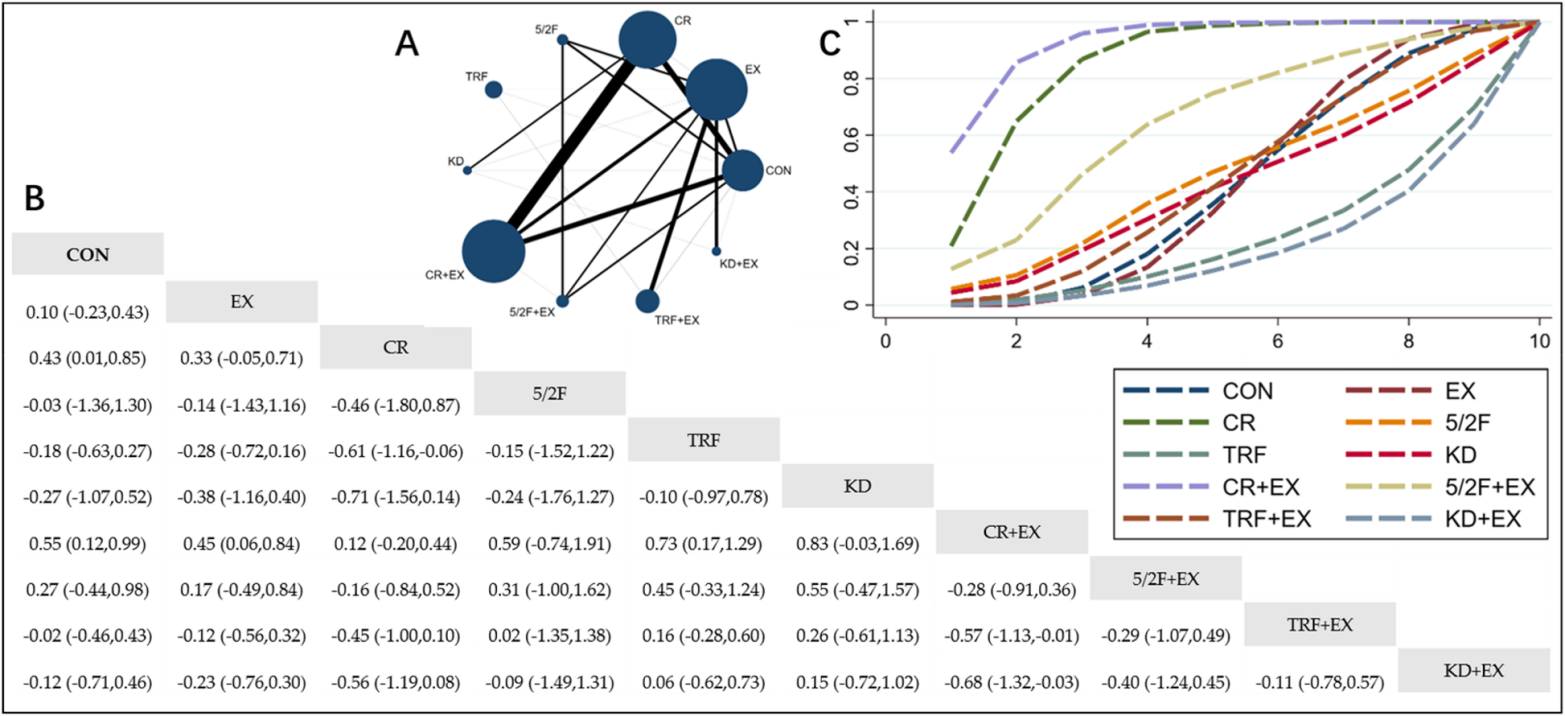
Network Meta-Analysis of TC: Network Plot, League Table, and SUCRA Plot. **A** presents the Network Plot. The size of the nodes is proportional to the sample size of each intervention, and the thickness of the lines corresponds to the number of available studies. **B** displays the pairwise comparison League Table, where the estimated effect size differences (SMD with 95% CI) represent the difference between the intervention on the top and the intervention on the right. **C** illustrates the SUCRA Plot, where the size of the area under the curve indicates the effectiveness of each intervention

The Network Plot, League Table, and SUCRA Plot for the effects of different interventions on TC are presented in Fig. 3.

As shown in Fig. 3A, most of the included studies mainly compared the effects of CR + EX versus CR or EX alone. The groups involving CR + EX, CR, EX, and CON had the largest number of participants. Figure 3B summarizes the estimated effect size differences (SMD with 95% CI) for pairwise comparisons of the 10 intervention methods. Compared to the CON group, both the CR group and the CR + EX group exhibited significant reductions in TC, while TC reductions in other groups were not significant. Figure 3C shows the SUCRA ranking of the 10 intervention methods, with the area under the curve representing the effectiveness of each intervention. A larger area under the curve indicates a higher ranking of effectiveness. For TC reduction, the ranking of the 10 interventions is as follows: CR + EX > CR > 5/2F + EX > 5/2F > TRF + EX > EX > CON > KD > TRF > KD + EX.

#### The effect of different interventions on HDL

**Fig. 4:**
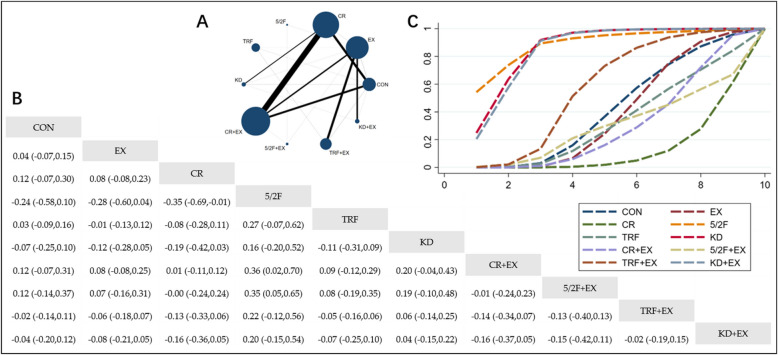
Network Meta-Analysis of HDL: Network Plot, League Table, and SUCRA Plot. **A** presents the Network Plot. The size of the nodes is proportional to the sample size of each intervention, and the thickness of the lines corresponds to the number of available studies. **B** displays the pairwise comparison League Table, where the estimated effect size differences (SMD with 95% CI) represent the difference between the intervention on the top and the intervention on the right. **C** illustrates the SUCRA Plot, where the size of the area under the curve indicates the effectiveness of each intervention

The Network Plot, League Table, and SUCRA Plot for the effects of different interventions on HDL are presented in Fig. 4.

As shown in Fig. 4A, most studies mainly investigated the effects of CR alone, TRF alone, and their combinations with exercise. The groups involving CR + EX, CR, EX, CON, TRF + EX, and TRF had the largest number of participants. Figure 4B summarizes the estimated effect size differences (SMD with 95% CI) for pairwise comparisons of the 10 intervention methods. Compared to the CON group, none of the 9 intervention groups demonstrated significant changes in HDL levels, though there was a trend toward HDL improvement in the 5/2F, KD, TRF + EX, and KD + EX groups. Figure 4C presents the SUCRA rankings for the 10 interventions, with the area under the curve representing the effectiveness of each intervention. A larger area under the curve indicates a higher ranking of effectiveness. The ranking of the 10 interventions for increasing HDL is as follows: 5/2F > KD > KD + EX > TRF + EX > CON > EX > TRF > 5/2F + EX > CR + EX > CR.

#### The effect of different interventions on LDL

**Fig. 5:**
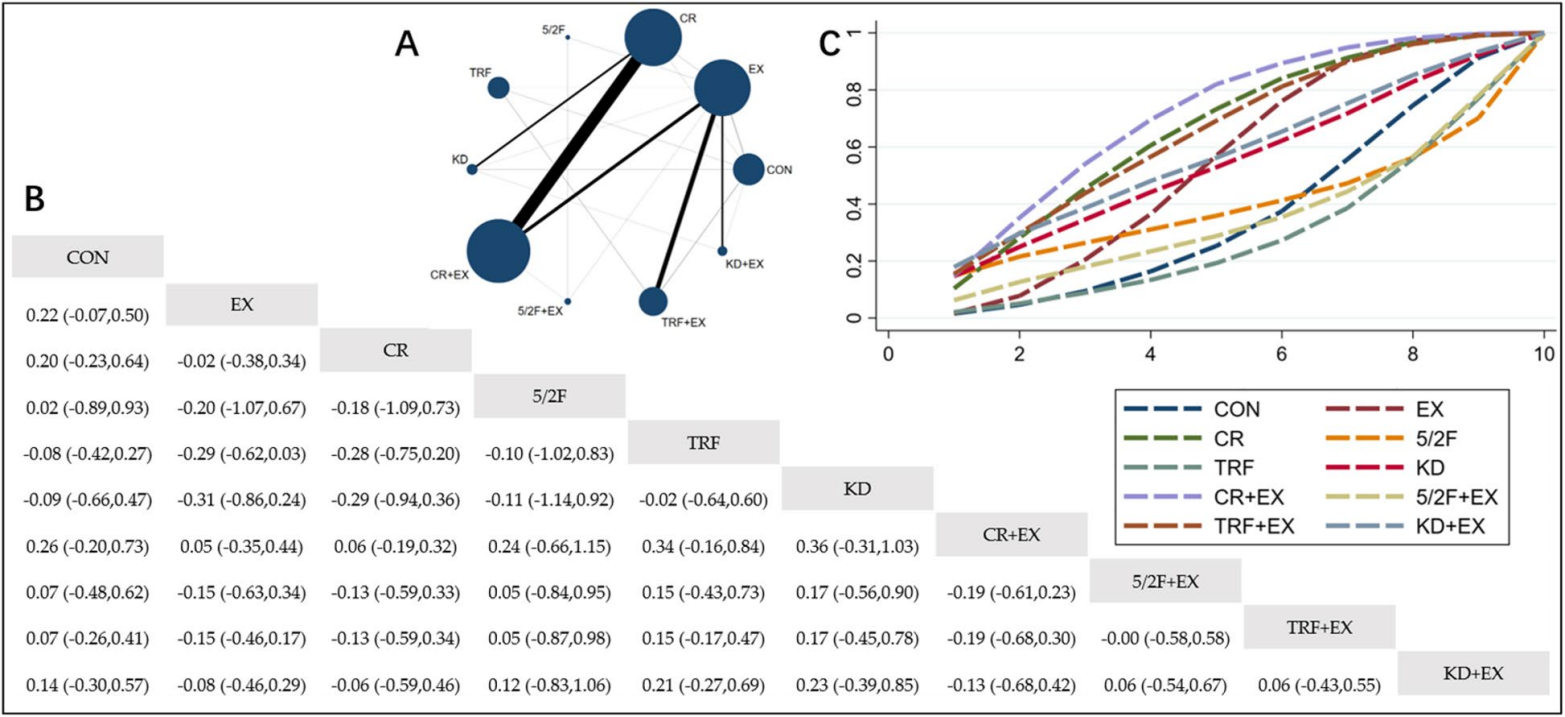
Network Meta-Analysis of LDL: Network Plot, League Table, and SUCRA Plot. **A** presents the Network Plot. The size of the nodes is proportional to the sample size of each intervention, and the thickness of the lines corresponds to the number of available studies. **B** displays the pairwise comparison League Table, where the estimated effect size differences (SMD with 95% CI) represent the difference between the intervention on the top and the intervention on the right. **C** illustrates the SUCRA Plot, where the size of the area under the curve indicates the effectiveness of each intervention

The Network Plot, League Table, and SUCRA Plot for the effects of different interventions on LDL are shown in Fig. 5.

As illustrated in Fig. 5A, most studies mainly investigated the effects of calorie restriction alone, time-restricted feeding alone, and their combinations with exercise interventions. The groups involving CR + EX, CR, EX, CON, TRF + EX, and TRF had the largest number of participants. Figure 5B summarizes the estimated effect size differences (SMD with 95% CI) for pairwise comparisons of the 10 intervention methods. Compared to the CON group, none of the 9 intervention groups showed significant changes in LDL levels, although there was a downward trend in LDL in the EX, CR, 5/2F, CR + EX, 5/2F + EX, TRF + EX, and KD + EX groups. Figure 5C presents the SUCRA rankings for the 10 interventions, with the area under the curve representing the effectiveness of each intervention. A larger area under the curve indicates a higher ranking of effectiveness. The ranking of the 10 interventions for lowering LDL is as follows: CR + EX > CR > TRF + EX > KD + EX > EX > KD > 5/2F > CON > 5/2F + EX > TRF.

#### The effect of different interventions on SBP

**Fig. 6:**
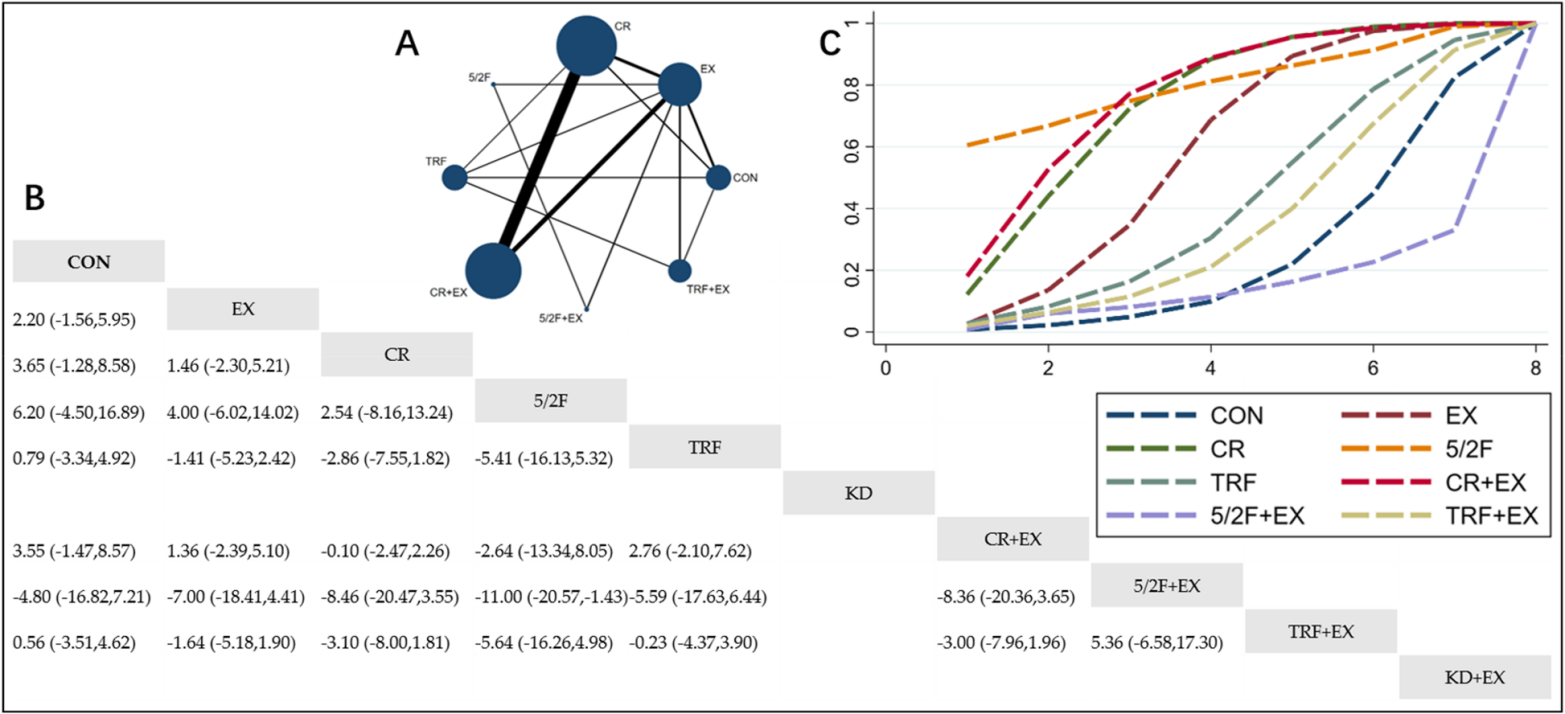
Network Meta-Analysis of SBP: Network Plot, League Table, and SUCRA Plot. **A** presents the Network Plot. The size of the nodes is proportional to the sample size of each intervention, and the thickness of the lines corresponds to the number of available studies. **B** displays the pairwise comparison League Table, where the estimated effect size differences (SMD with 95% CI) represent the difference between the intervention on the top and the intervention on the right. **C** illustrates the SUCRA Plot, where the size of the area under the curve indicates the effectiveness of each intervention

The Network Plot, League Table, and SUCRA Plot for the effects of different interventions on SBP are shown in Fig. 6.

As shown in Fig. 6A, most studies mainly investigated the effects of calorie restriction alone, time-restricted feeding alone, and their combinations with exercise. No relevant studies were identified for KD + EX or KD alone. The groups involving CR + EX, CR, EX, CON, TRF + EX, and TRF had the largest number of participants. Figure 6B summarizes the estimated effect size differences (SMD with 95% CI) for pairwise comparisons of the 8 intervention methods. Compared to the CON group, none of the 7 intervention groups showed significant changes in SBP, although there was a downward trend in SBP in the EX, CR, 5/2F, TRF, CR + EX, and TRF + EX groups. Figure 6C presents the SUCRA rankings for the 8 interventions, with the area under the curve representing the effectiveness of each intervention. A larger area under the curve indicates a higher ranking of effectiveness. The ranking of the 8 interventions for lowering SBP is as follows: 5/2F > CR + EX > CR > EX > TRF > TRF + EX > CON > 5/2F + EX.

#### The effect of different interventions on DBP

**Fig. 7:**
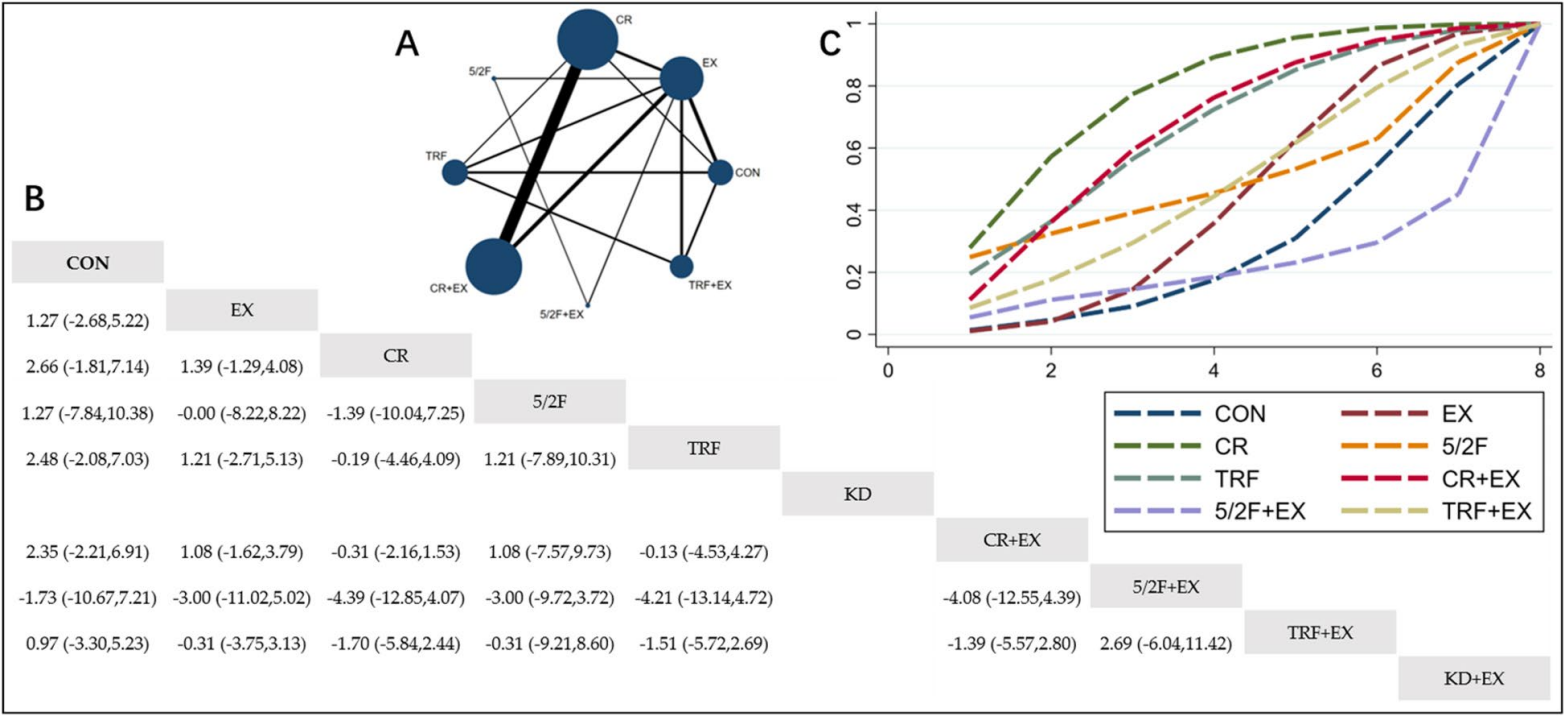
Network Meta-Analysis of DBP: Network Plot, League Table, and SUCRA Plot. **A** presents the Network Plot. The size of the nodes is proportional to the sample size of each intervention, and the thickness of the lines corresponds to the number of available studies. **B** displays the pairwise comparison League Table, where the estimated effect size differences (SMD with 95% CI) represent the difference between the intervention on the top and the intervention on the right. **C** illustrates the SUCRA Plot, where the size of the area under the curve indicates the effectiveness of each intervention

The Network Plot, League Table, and SUCRA Plot for the effects of different interventions on DBP are shown in Fig. 7.

As shown in Fig. 7A, most studies mainly investigated the effects of calorie restriction alone, time-restricted feeding alone, and their combinations with exercise. No relevant studies were identified for KD + EX or KD alone. The groups involving CR + EX, CR, EX, CON, TRF + EX, and TRF had the largest number of participants. Figure 7B summarizes the estimated effect size differences (SMD with 95% CI) for pairwise comparisons of the 8 intervention methods. In comparison with the CON group, none of the 7 intervention groups demonstrated statistically significant changes in DBP, although a downward trend in DBP was observed in the EX, CR, 5/2F, TRF, CR + EX, and TRF + EX groups. Figure 7C presents the SUCRA rankings for the 8 interventions, with the area under the curve representing the effectiveness of each intervention. A larger area under the curve indicates a higher ranking of effectiveness. The ranking of the 8 interventions for lowering DBP is as follows: CR > CR + EX > TRF > 5/2F > TRF + EX > EX > CON > 5/2F + EX.

#### The effect of different interventions on HDL + LDL

**Fig. 8:**
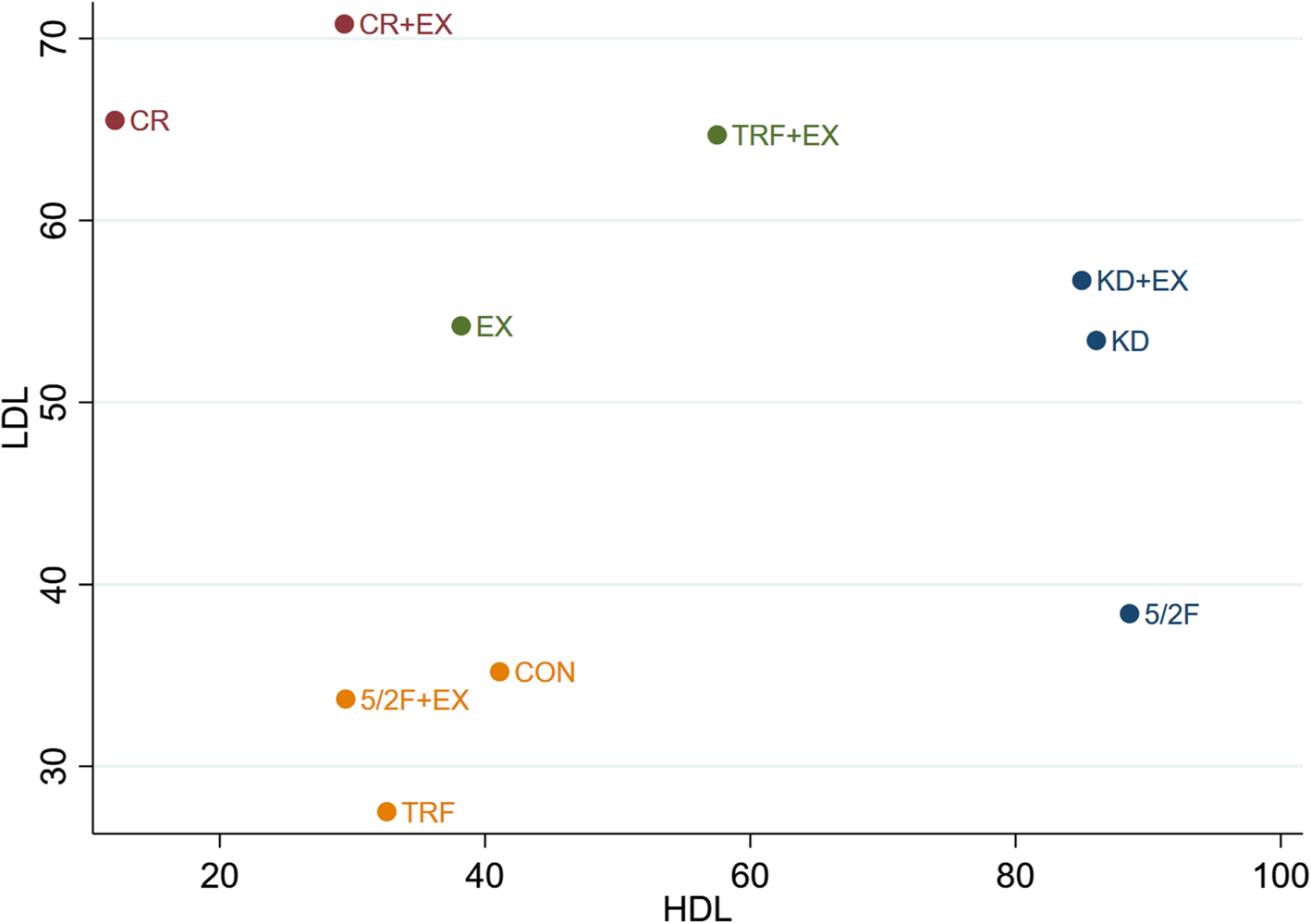
The Clustered Ranking Plot of HDL + LDL. The horizontal axis represents the effect on increasing HDL, and the vertical axis represents the effect on reducing LDL, with points in the top right representing the best effects

The Clustered Ranking Plot for the effects of different interventions on HDL + LDL is shown in Fig. 8.

As shown in Fig. 8, KD + EX, KD, and TRF + EX interventions had the best effects in reducing LDL and increasing HDL. In contrast, TRF, 5/2F + EX, and CON had the poorest effects in reducing LDL and increasing HDL. Although CR + EX and CR showed good effects in reducing LDL, they were less effective in increasing HDL. However, while 5/2F had poor effects in reducing LDL, it was the most effective in increasing HDL.

#### The effect of different interventions on SBP + DBP

**Fig. 9:**
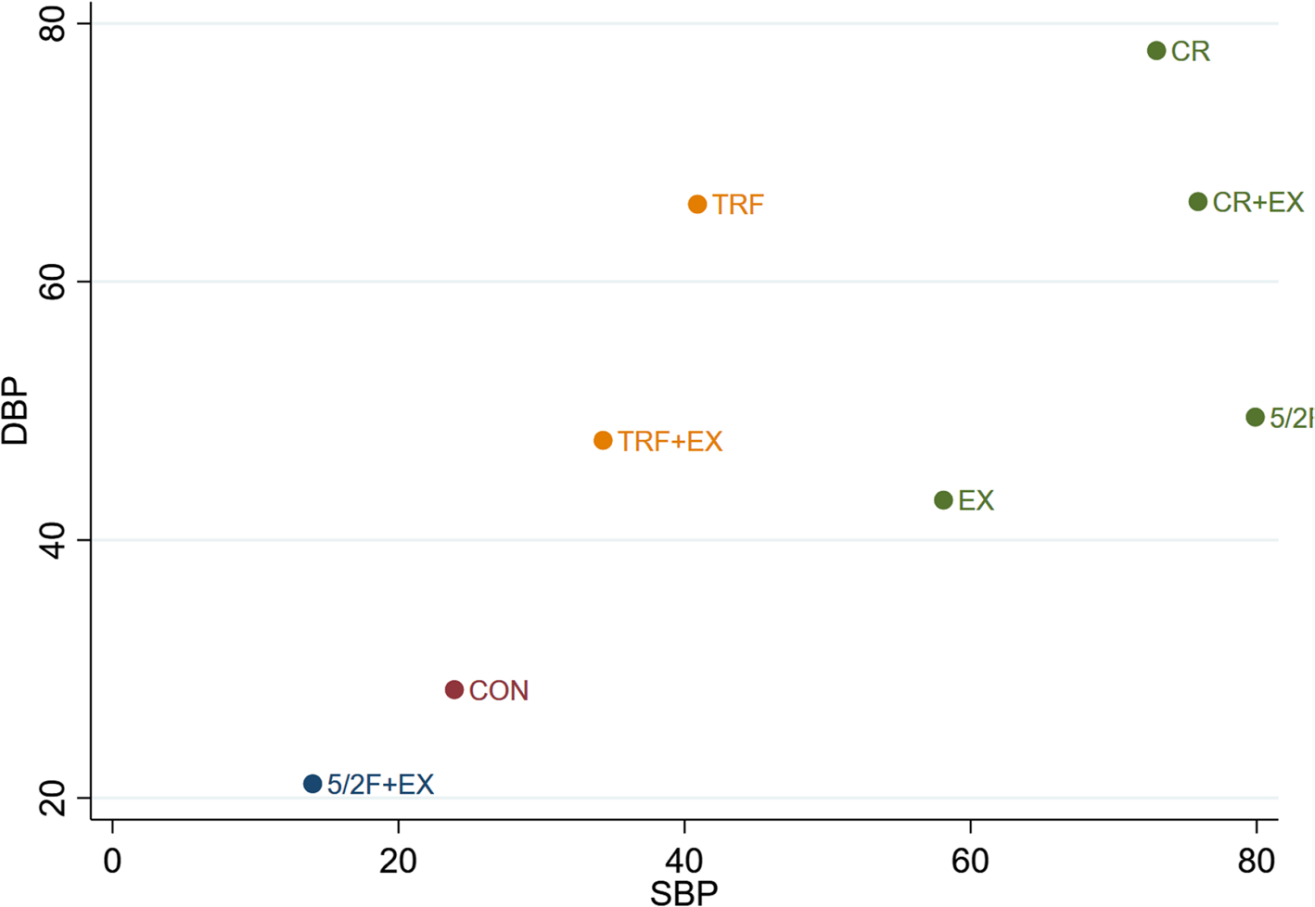
The Clustered Ranking Plot of SBP + DBP. The horizontal axis represents the effect on reducing SBP, and the vertical axis represents the effect on reducing DBP, with points in the top right representing the best effects

The Clustered Ranking Plot for the effects of different interventions on SBP + DBP is shown in Fig. 9.

As shown in Fig. 9, all interventions followed a linear trend, indicating that the effect of interventions on SBP and DBP was generally consistent. CR and CR + EX had the best effects in reducing both SBP and DBP, while the effect of 5/2F + EX on reducing SBP and DBP was even worse than CON.

## Discussion

Our network meta-analysis (NMA) included 37 controlled trials assessing the effects of exercise, dietary interventions, and their combination on cardiovascular health indicators. Based on the included studies, we ranked the effects of 10 interventions —CON, EX, CR, 5/2F, TRF, KD, CR + EX, 5/2F + EX, TRF + EX, and KD + EX— on key cardiovascular health markers. Our findings indicate that CR and CR + EX had the most positive effects on cardiovascular health. While these interventions led to a reduction in HDL, they significantly lowered TC, TG, and LDL levels. Notably, 5/2F + EX had a negative impact on reducing SBP and DBP, being less effective than the CON group. Additionally, we observed significant variation in the effects of different dietary and exercise interventions on various cardiovascular indicators. For instance, while CR and CR + EX significantly reduced LDL, they had minimal effect on increasing HDL. Conversely, 5/2F had a notable effect on increasing HDL, while it was less effective at lowering LDL.

The results of this NMA align with our previous studies, which identified CR + EX and CR as the most effective interventions for improving body composition and cardiovascular health [71]. There is a well-established correlation between body composition and cardiovascular health indicators: higher body fat percentages are associated with elevated levels of TG, TC, and LDL [11, 50]. Additionally, a higher body fat percentage often correlates with hypertension [73]. In terms of cardiovascular health, the effects of CR + EX and CR are significantly greater than those of EX alone, indicating that the primary benefits of CR + EX stem from the calorie restriction component. CR typically involves limiting total caloric intake, which often results in the avoidance of high-fat foods. To increase satiety, individuals may opt for protein-rich meats and vitamin-rich fruits and vegetables. In the case of TRF and 5/2F, individuals may exhibit compensatory eating behaviors during feeding periods following fasting, often with a stronger inclination toward high-calorie foods, increasing the intake of carbohydrates and fats. In contrast, the KD involves consuming large amounts of fat to stimulate ketosis, with excessive fat intake being the primary cause of elevated plasma TG, TC, and LDL. Among the 10 intervention methods, only CR + EX and CR indirectly limit fat intake, which is why they are the most effective interventions for reducing blood lipids. The relationship between obesity and hypertension is well-documented, with estimates indicating that obesity accounts for 65–78% of primary hypertension cases [73]. Some studies have also indicated that blood pressure may be influenced by body weight and fat percentage. Previous research has shown that CR + EX and CR are the most effective interventions for reducing body weight and fat percentage [71]. Given that body weight and fat percentage can influence blood pressure, this partly explains the effectiveness of CR + EX and CR in lowering blood pressure. Additionally, CR may help reduce hypertension by improving vascular function. One study demonstrated that CR enhances vascular health by targeting multiple pathways related to inflammation, ROS production, extracellular matrix deposition, and nitric oxide synthesis, thereby lowering blood pressure [74]. Furthermore, research indicates that short-term, mild CR improves endothelial function and lowers blood pressure in obese patients by activating the AMPK-PI3K-Akt-eNOS pathway [24]. These findings align with the results of this study. Therefore, for lowering blood lipids and blood pressure, CR is the most effective approach, and CR + EX offers even greater benefits.

Surprisingly, the results of this study show that the combination of 5/2F + EX and TRF was less effective than CON in promoting cardiovascular health. Specifically, 5/2F + EX was less effective than CON in increasing HDL, lowering LDL, and reducing both SBP and DBP. Our findings suggest that while 5/2F alone has positive health effects in sedentary individuals, ranking relatively high, combining it with exercise may overwhelm the body's capacity or disrupt the metabolic balance achieved through 5/2F. This could ultimately result in negative effects. This conclusion aligns with previous research [69]. Upon examining the original data, we found that the comparisons between 5/2F + EX and CON for HDL, LDL, SBP, and DBP were based on indirect comparisons. Although no direct comparison data were available, the results of these indirect comparisons should still be considered. However, since results from indirect comparisons are more susceptible to variability across studies, further experimental research is required to validate these findings. For TRF and CON, two studies provided direct comparisons for HDL, LDL, TG, and TC, which were largely consistent with the NMA results. The fasting duration in TRF may have been insufficient, potentially causing feeding to begin before the body could undergo adaptive metabolic changes. Additionally, shorter fasting durations might have had a greater impact on appetite than on energy metabolism, resulting in compensatory overeating of sugars and fats. This increased overall caloric intake, ultimately raising LDL, TG, and TC levels [75]. Therefore, for individuals seeking non-pharmacological methods to reduce blood lipids, the 5/2F + EX and TRF interventions should be avoided. Similarly, for those aiming to lower blood pressure without medication, the 5/2F + EX intervention is not recommended.

Our study also uncovered an interesting phenomenon: 5/2F + EX was less effective in lowering blood pressure than 5/2F alone. Similarly, TRF + EX was less effective than TRF alone, and KD + EX was less effective than KD alone in reducing TG and TC. These results suggest that, in terms of maintaining cardiovascular health, not all dietary interventions combined with exercise lead to better outcomes. However, these findings are specific to certain cardiovascular health indicators and do not offer definitive guidance for other areas of health. For blood pressure reduction, both intermittent fasting methods (5/2F and TRF) demonstrated positive effects; However, when combined with exercise, they produced negative outcomes. This aligns with previous research, which found that while the combination of EX and IF improved body composition, it did not enhance cardiometabolic health markers compared to EX or IF alone [69]. Therefore, combined interventions may be effective for reducing weight and fat, but they do not provide additional or synergistic benefits for other cardiometabolic health markers. This could be because individual dietary or exercise interventions fall within an optimal intensity range for promoting cardiovascular health. However, combining these interventions may exceed this range, potentially leading to negative effects or even harm to cardiovascular health. The underlying reasons, however, require further investigation. Similarly, KD + EX was less effective in reducing TG and TC compared to KD alone, which is consistent with previous studies showing that KD + EX was less effective than KD alone in reducing body fat percentage [71]. This could be because exercise, while in a ketogenic state, promotes fat absorption and storage. Since fat is the most efficient energy source in ketosis, the body may accumulate more fat with regular exercise compared to KD alone. Therefore, not all dietary interventions are suitable for combination with exercise.

In the results of this NMA, the trend of changes in HDL concentration was opposite to that of the other five outcome indicators. While CR + EX and CR have been shown in numerous studies to significantly improve cardiovascular health, this study found that CR + EX and CR were less effective at increasing HDL concentrations compared to other interventions. In fact, they even showing lower HDL levels than the control group. HDL plays a critical role in cardiovascular health. Circulating HDL is a heterogeneous mixture of lipoprotein particles, which vary in size, density, and composition [5]. These lipoprotein-associated enzymes esterify cholesterol into cholesteryl ester (CE). Due to its hydrophobic nature, migrates from the HDL particle surface to the core. This process restricts the return of cholesterol to peripheral tissues via passive diffusion, allowing CE to be transported by HDL to the liver for metabolism [76]. Additionally, several HDL-associated enzymes, including paraoxonase 1 (PON1) and lecithin-cholesterol acyltransferase (LCAT), exhibit antioxidant properties that may prevent LDL oxidation. LCAT hydrolyzes oxidized short-chain phospholipids [77]. Some studies suggest that a low intake of fats and oils increases the risk of insufficient vitamin E and essential fatty acids, which may reduce HDL levels [48, 78]. Since CR typically involves caloric restriction, it often leads to lower fat and oil intake to enhance satiety, which may explain the findings of this study. Therefore, measuring HDL concentration alone is insufficient to fully reflect the cholesterol-clearing capacity of plasma.

In the quest for indicators that more accurately reflect the cholesterol-clearing capacity of plasma, a large cohort study found that HDL concentration alone does not predict the incidence of adverse cardiovascular events. The study provided evidence that cholesterol efflux capacity, rather than HDL levels, is the key function of HDL lipoprotein particles [79]. Several studies have employed lipid ratios to reflect the cholesterol-clearing capacity of plasma, such as the triglyceride-to-HDL ratio (TG/HDL) and the non-high-density lipoprotein cholesterol to high-density lipoprotein cholesterol ratio [49, 80, 81]. These ratios have been used in the risk assessment of cardiovascular diseases [82], as well as in studies involving the digestive [83, 84] and urinary systems [85]. These ratios represent the relative concentration of pro-atherosclerotic lipid particles to anti-atherosclerotic lipid particles. They may offer greater diagnostic and predictive advantages than traditional lipid parameters [86].

Our study also found that none of the interventions resulted in positive changes across all six cardiovascular health indicators. For example, CR and CR + EX significantly lowered LDL, TG, DBP, and SBP, but had minimal effect on HDL levels. This emphasizes that a single outcome indicator cannot fully represent cardiovascular health, and a comprehensive assessment of all indicators is necessary. This involves two key aspects: first, no single outcome indicator alone can cause ultimate cardiovascular damage [87]. Second, changes in the concentration of a single indicator may involve other mechanisms, so its results should not be interpreted in isolation. For instance, one study found that a notable side effect of KD is a sharp increase in TG and LDL levels during the initial months, but this does not necessarily indicate a heightened risk of atherosclerosis. The rise in LDL levels associated with KD is accompanied by an increase in the size and volume of LDL particles, which may actually reduce the likelihood of these particles causing atherosclerosis [58]. Therefore, even if CR + EX and CR are less effective at increasing HDL, this does not diminish their leading role in promoting overall cardiovascular health.

### Strengths and limitations

A notable limitation of this study is the lack of literature measuring systolic and diastolic blood pressure in KD interventions, making it impossible to assess the effects of KD on blood pressure reduction. Additionally, most of the included literature focuses on CR and EX, while studies on other interventions are limited. This means that the final results for these interventions are more susceptible to the influence of indirect comparisons. Furthermore, using HDL concentration as a sole indicator to reflect the cholesterol-clearing capacity of plasma is imprecise, yet many studies still regard an increase in HDL as a marker of cardiovascular health improvement. This has impacted the assessment of how the interventions in this study affect blood lipid profiles. Additionally, this study only controlled for exercise intensity and did not group by exercise type. This may lead to some heterogeneity between different exercise types.

## Conclusions

Among the nine intervention methods, CR and CR + EX had the most positive effects on cardiovascular health indicators. In contrast, 5/2F + EX ranked relatively low, with its effects on many indicators even being worse than CON.

## Supplementary Information

Supplementary Material 1. File S1: Searching Strategy; Figure S1: Risk of bias summary; Figure S2: Risk of bias graph; Table S1: Characteristic of included trials. References [88–124] are cited in the Supplementary Materials.

## Data Availability

No datasets were generated or analysed during the current study.
