# Supplementary material for: Effects of exercise combined with different dietary interventions on cardiovascular health a systematic review and network meta-analysis
Source: BMC Cardiovasc Disord. 2025 Mar 26;25:222. doi: 10.1186/s12872-025-04666-z (PMC11938602; doi:10.1186/s12872-025-04666-z)
Supplement: Supplementary file 1 — Supplementary Material 1. File S1: Searching Strategy; Figure S1: Risk of bias summary; Figure S2: Risk of bias graph; Table S1: Characteristic of included trials. References [88–124] are cited in the Supplementary Materials. [file 12872_2025_4666_MOESM1_ESM.docx]

Supplementary File S1. Searching Strategy

Supplementary Figures S1. Risk of bias summary

Supplementary Figures S2. Risk of bias graph

Supplementary Table S1. Characteristic of included trials.

Supplementary File S1. Searching Strategy

**PubMed**

"Exercise"[Mesh Terms] AND ("Caloric Restriction"[Mesh Terms] OR "diet, ketogenic"[Mesh Terms] OR "Intermittent Fasting"[Mesh Terms]) AND ("Body Weight"[Mesh Terms] OR "Body Mass Index"[Mesh Terms] OR "Triglycerides"[Mesh Terms] OR "lipoproteins, hdl"[Mesh Terms] OR "lipoproteins, ldl"[Mesh Terms] OR "Cholesterol"[Mesh Terms] OR "Blood Pressure"[Mesh Terms]) AND ("randomized controlled trial"[Publication Type] OR "randomized"[Title/Abstract] OR "placebo"[Title/Abstract] OR "RCT"[Title/Abstract])

**Web of Science**

#1

TS=（Exercise）OR TS=（Exercises）OR TS=（Physical Activity）OR TS=（Activities, Physical）OR TS=（Activity, Physical）OR TS=（Physical Activities）OR TS=（Exercise, Physical）OR TS=（Exercises, Physical）OR TS=（Physical Exercise）OR TS=（Physical Exercises）OR TS=（Acute Exercise）OR TS=（Acute Exercises）OR TS=（Exercise, Acute）OR TS=（Exercises, Acute）OR TS=（Exercise, Isometric）OR TS=（Exercises, Isometric）OR TS=（Isometric Exercises）OR TS=（Isometric Exercise）OR TS=（Exercise, Aerobic）OR TS=（Aerobic Exercise）OR TS=（Aerobic Exercises）OR TS=（Exercises, Aerobic）OR TS=（Exercise Training）OR TS=（Exercise Trainings）OR TS=（Training, Exercise）OR TS=（Trainings, Exercise）

#2

TS=（Caloric Restriction ）OR TS=（Restriction, Caloric）OR TS=（Calorie Restricted Diet）OR TS=（Calorie Restricted Diets）OR TS=（Diet, Calorie Restricted）OR TS=（Restricted Diet, Calorie）OR TS=（Caloric Restricted）OR TS=（Restricted, Caloric）OR TS=（Low-Calorie Diet）OR TS=（Diet, Low-Calorie）OR TS=（Low Calorie Diet）OR TS=（Low-Calorie Diets）

#3

TS=（Diet, Ketogenic）OR TS=（Ketogenic Diet）OR TS=（Diets, Ketogenic）OR TS=（Ketogenic Diets）

#4

TS=（Intermittent Fasting）OR TS=（Fasting, Intermittent）OR TS=（Meal Skipping）OR TS=（Skipping, Meal）OR TS=（Breakfast Skipping）OR TS=（Skipping, Breakfast）OR TS=（Time Restricted Fasting）OR TS=（Fasting, Time Restricted）OR TS=（Restricted Fastings, Time）OR TS=（Time Restricted Eating）OR TS=（Eating, Time Restricted）OR TS=（Time Restricted Feeding）OR TS=（Feeding, Time Restricted）OR TS=（Time Restricted Feedings）

#5

TS=（Body Weight）OR TS=（Body Weights）OR TS=（Weight, Body）OR TS=（Weights, Body）

#6

TS=（Body Mass Index）OR TS=（Index, Body Mass）OR TS=（Quetelet Index）OR TS=（Index, Quetelet）OR TS=（Quetelet's Index）OR TS=（Quetelets Index）

#7

TS=（Triglycerides）OR TS=（Triacylglycerols）OR TS=（Triacylglycerol）OR TS=（Triglyceride）

#8

TS=（Lipoproteins, HDL）OR TS=（HDL Lipoproteins）OR TS=（High-Density Lipoprotein）OR TS=（Lipoprotein, High-Density）OR TS=（High-Density Lipoproteins）OR TS=（High Density Lipoproteins）OR TS=（Lipoproteins, High-Density）OR TS=（alpha-Lipoproteins）OR TS=（alpha Lipoproteins）OR TS=（Heavy Lipoproteins）OR TS=（Lipoproteins, Heavy）OR TS=（High Density Lipoprotein）OR TS=（Density Lipoprotein, High）OR TS=（Lipoprotein, High Density）OR TS=（alpha-Lipoprotein）OR TS=（alpha Lipoprotein）OR TS=（alpha-1 Lipoprotein）

#9

TS=（Lipoproteins, LDL）OR TS=（LDL Lipoproteins）OR TS=（beta-Lipoprotein）OR TS=（beta Lipoprotein）OR TS=（Low-Density Lipoproteins）OR TS=（Lipoproteins, Low-Density）OR TS=（Low Density Lipoproteins）OR TS=（beta-Lipoproteins）OR TS=（beta Lipoproteins）OR TS=（Low-Density Lipoprotein）OR TS=（Lipoprotein, Low-Density）OR TS=（Low Density Lipoprotein）OR TS=（LDL-2）OR TS=（LDL2）OR TS=（Low-Density Lipoprotein 2）OR TS=（Low Density Lipoprotein 2）OR TS=（LDL(2)）OR TS=（LDL-1）OR TS=（LDL1）OR TS=（Low-Density Lipoprotein 1）OR TS=（Low Density Lipoprotein 1）OR TS=（LDL(1)）

#10

TS=（Cholesterol）OR TS=（Epicholesterol）

#11

TS=（Blood Pressure）OR TS=（Pressure, Blood）OR TS=（Diastolic Pressure）OR TS=（Pressure, Diastolic）OR TS=（Pulse Pressure）OR TS=（Pressure, Pulse）OR TS=（Systolic Pressure）OR TS=（Pressure, Systolic）OR TS=（Pressures, Systolic）

#12

#2 OR #3 OR #4

#13

#5 OR #6 OR #7 OR #8 OR #9 OR #10 OR #11

#14

TS=（randomized controlled trial）OR TS=（randomized）OR TS=（placebo）

#15

#1 AND #12 AND #13 AND #14

**Embase**

#1

'exercise'/exp

#2

'caloric restriction'/exp OR 'ketogenic diet'/exp OR 'intermittent fasting'/exp

#3

'body weight'/exp OR 'body mass'/exp OR 'triacylglycerol'/exp OR 'high density lipoprotein'/exp OR 'low density lipoprotein'/exp OR 'cholesterol'/exp OR 'blood pressure'/exp

#4

'randomized controlled trial'/exp

#5

#1 AND #2 AND #3 AND #4

**Cochrane Library**

#1 Mesh descriptor: [Exercise] explode all trees

#2 Mesh descriptor: [Caloric Restriction] explode all trees

#3 Mesh descriptor: [Diet, Ketogenic] explode all trees

#4 Mesh descriptor: [Intermittent Fasting] explode all trees

#5 Mesh descriptor: [Body Weight] explode all trees

#6 Mesh descriptor: [Body Mass Index] explode all trees

#7 Mesh descriptor: [Triglycerides] explode all trees

#8 Mesh descriptor: [Lipoproteins, HDL] explode all trees

#9 Mesh descriptor: [Lipoproteins, LDL] explode all trees

#10 Mesh descriptor: [Cholesterol] explode all trees

#11 Mesh descriptor: [Blood Pressure] explode all trees

#12 #2 OR #3 OR #4

#13 #5 OR #6 OR #7 OR #8 OR #9 OR #10 OR #11

#14 #1 AND #12 AND #13


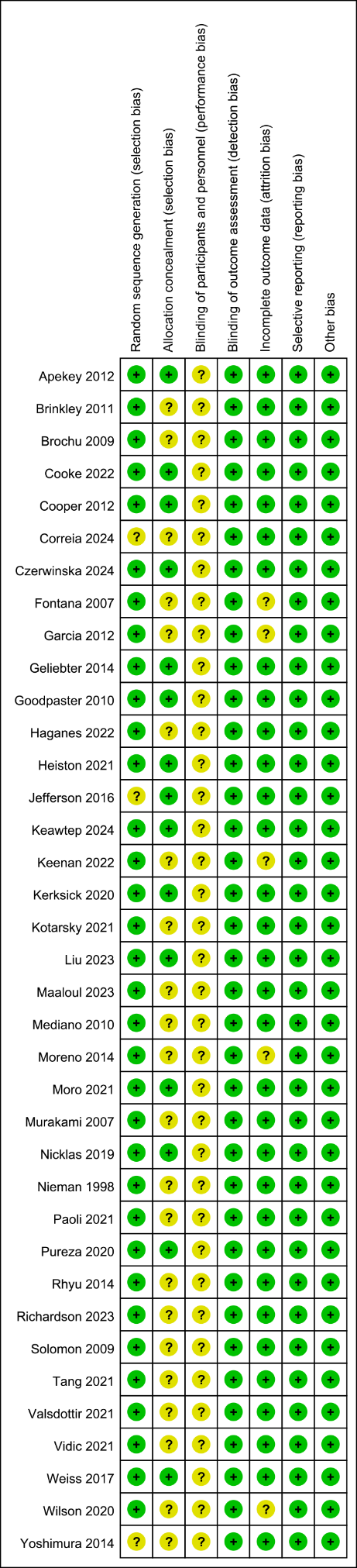


Figure S1. Risk of bias summary


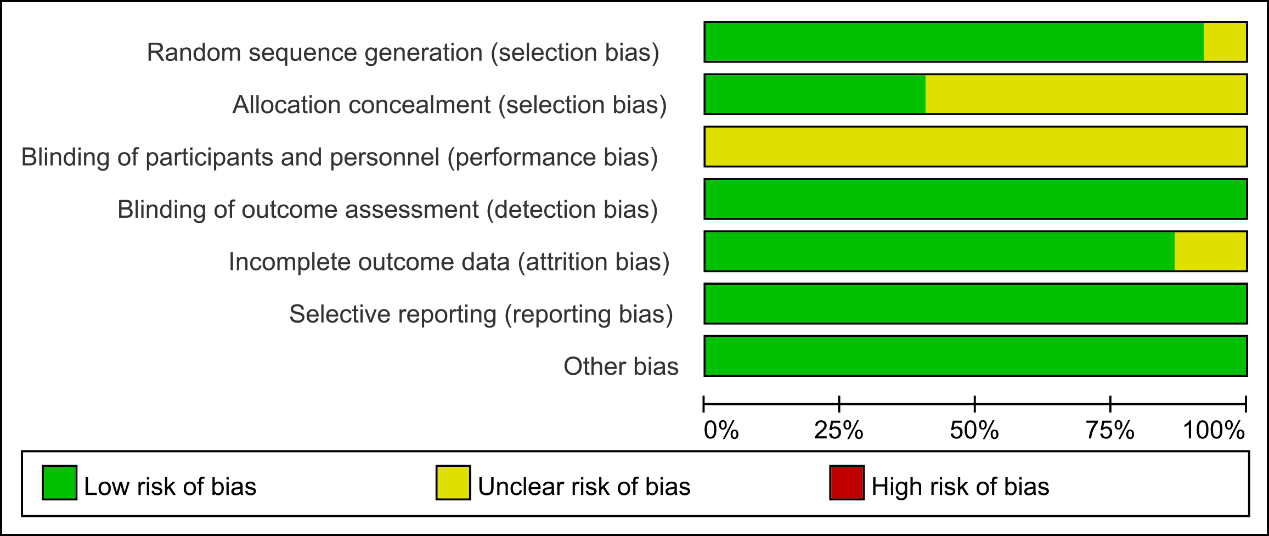


Figure S2. Risk of bias graph

Table S1. Characteristic of included trials.

|  | Study | Sample Characteristics | Type of intervention | Duration | main indicators |
| --- | --- | --- | --- | --- | --- |
| 1 | Apekey 2012[88] | Obese adults, 18 years old | CR, CR+EX | 8w | TG, TC, HDL, LDL, SBP, DBP |
| 2 | Brinkley 2011[89] | Obese postmenopausal women, 58 years old | CR, CR+EX | 20w | TG, TC, HDL, LDL, SBP, DBP |
| 3 | Brochu 2009[90] | Obese postmenopausal women, 58 years old | CR, CR+EX | 6m | TG, TC, HDL, LDL, SBP, DBP |
| 4 | Cooke 2022[91] | Obese adults, 35.4 years old | EX, 5/2F, 5/2F+EX | 16w | TG, TC, HDL, LDL, SBP, DBP |
| 5 | Cooper 2012[92] | Severely obese adults, 33 years old | CR, CR+EX | 6m | TG, TC, HDL, LDL, SBP, DBP |
| 6 | Correia 2024[93] | Healthy male amateur runner, 23.7 years old | EX, TRF+EX | 4w | TG, TC, HDL, LDL |
| 7 | Czerwińska 2024[94] | Obese women, 21-85 years old | CON, TRF+EX | 12w | TG, TC, HDL, LDL |
| 8 | Fontana 2007[95] | Non-obese subjects, 57 years old | EX, CR, CON | 12m | TG, TC, HDL, LDL, SBP, DBP |
| 9 | Garcia 2012[96] | Obese women, 50 years old | CON, CR, CR+EX | 16w | TG, TC, HDL |
| 10 | Geliebter 2014[97] | Obese adults, 35 years old | CR+EX, CR | 8w | TG, HDL, LDL, SBP, DBP |
| 11 | Goodpaster 2010[98] | Severely obese adults, 46 years old | CR+EX, CR | 6m | TG, TC, HDL, SBP, DBP |
| 12 | Haganes 2022[99] | Obese women, 36 years old | CON, TRF, EX, TRF+EX | 7w | TG, TC, HDL, LDL, SBP, DBP |
| 13 | Heiston 2021[100] | Obese women, 46 years old | CR, CR+EX | 2w | TC, HDL, LDL, SBP, DBP |
| 14 | Jefferson 2016[101] | Obese adults, 68 years old | EX, CR+EX | 5m | SBP, DBP |
| 15 | Keawtep 2024[60] | Obese postmenopausal women, 53 years old | CON, 5/2F, EX, 5/2F+EX | 3m | TG, TC |
| 16 | Keenan 2022[102] | Healthy men and women, 24 years old | 5/2F+EX, CR+EX | 12w | TG, TC, HDL, LDL |
| 17 | Kerksick 2020[103] | Postmenopausal women, 51 years old | EX, CR+EX | 14w | TG, TC, HDL, LDL, SBP, DBP |
| 18 | Kotarsky 2021[104] | Obese adults, 44 years old | EX, TRF+EX | 8w | TC, HDL |
| 19 | Liu 2023[105] | Hidden obesity female college students, 20 years old | CON, TRF, EX, TRF+EX | 8w | TG, TC, HDL, LDL, SBP, DBP |
| 20 | Maaloul 2023[106] | Obese men, 32 years old | TRF, TRF+EX | 1m | TG, TC, HDL, LDL |
| 21 | Mediano 2010[107] | Non-obese women, 37 years old | CR, CR+EX | 12M | TG, TC, HDL, LDL |
| 22 | Moreno 2014[108] | Obese adults, 45 years old | KD, CR | 12m | TG, TC, HDL, LDL |
| 23 | Moro 2021[109] | Endurance training experience, 30 years old | TRF+EX, EX | 12m | TG, TC, HDL, LDL |
| 24 | Murakami 2007[110] | Obese adults, 50 years old | CR, CR+EX | 12w | TG, TC, HDL, LDL, SBP, DBP |
| 25 | Nicklas 2019[111] | Obesity in older men and women, 69 years old | EX, CR+EX | 20w | TG, TC, HDL, LDL |
| 26 | Nieman 1998[112] | Obese women, 45 years old | CON, EX, CR, CR+EX | 12w | TG, TC |
| 27 | Paoli 2021[113] | Male bodybuilder, 27 years old | KD+EX, EX | 8w | TG, TC, HDL, LDL |
| 28 | Pureza 2020[114] | Obese women, 30 years old | CR, TRF | 3w | SBP, DBP |
| 29 | Rhyu 2014[115] | Healthy men and women, 18 years old | KD+EX, EX | 3w | BW, BMI, FM, FFM |
| 30 | Richardson 2023[116] | Male runner, 29 years old | EX, TRF+EX | 4w | TG, TC, HDL, LDL, SBP, DBP |
| 31 | Solomon 2009[117] | Obesity in older men and women, 66 years old | EX, CR+EX | 12w | TG, TC |
| 32 | Tang 2021[118] | Undergraduate students, 20 years old | CR, EX, CR+EX | 8w | TG, TC, HDL, LDL, SBP, DBP |
| 33 | Valsdottir 2021[119] | Obese women, 40 years old | CON, KD, EX, KD+EX | 10w | TG, TC, HDL, LDL |
| 34 | Vidic 2021[120] | Resistance Training for Men, 42 years old | KD+EX, EX | 4w | TG, TC, HDL, LDL |
| 35 | Weiss 2017[121] | Obese adults, 57 years old | CR, EX, CR+EX | 17w | SBP, DBP |
| 36 | Wilson 2020[122] | Resistance Training for Men, 22 years old | KD+EX, EX | 10w | TG, TC, HDL |
| 37 | Yoshimura 2014[123] | Obese adults, 55 years old | CR, CR+EX | 12w | TG, TC, HDL, LDL, SBP, DBP |

CON=The group without any intervention， EX=Exercise， CR=Caloric Restriction， 5/2F=5/2 Intermittent Fasting， TRF=Time-restricted Fasting， KD=Ketogenic Diet。 BW=Body Weight, BMI=Body Mass Index, FM%=Fat Percentage, FM=Fat Mass, FFM=Fat Free Mass, TG= Triglycerides, HDL= High-density Lipoprotein, LDL= Low-density Lipoprotein, TC= Total Cholesterol, SBP= Systolic Blood Pressure, DBP= Diastolic Blood Pressure.
